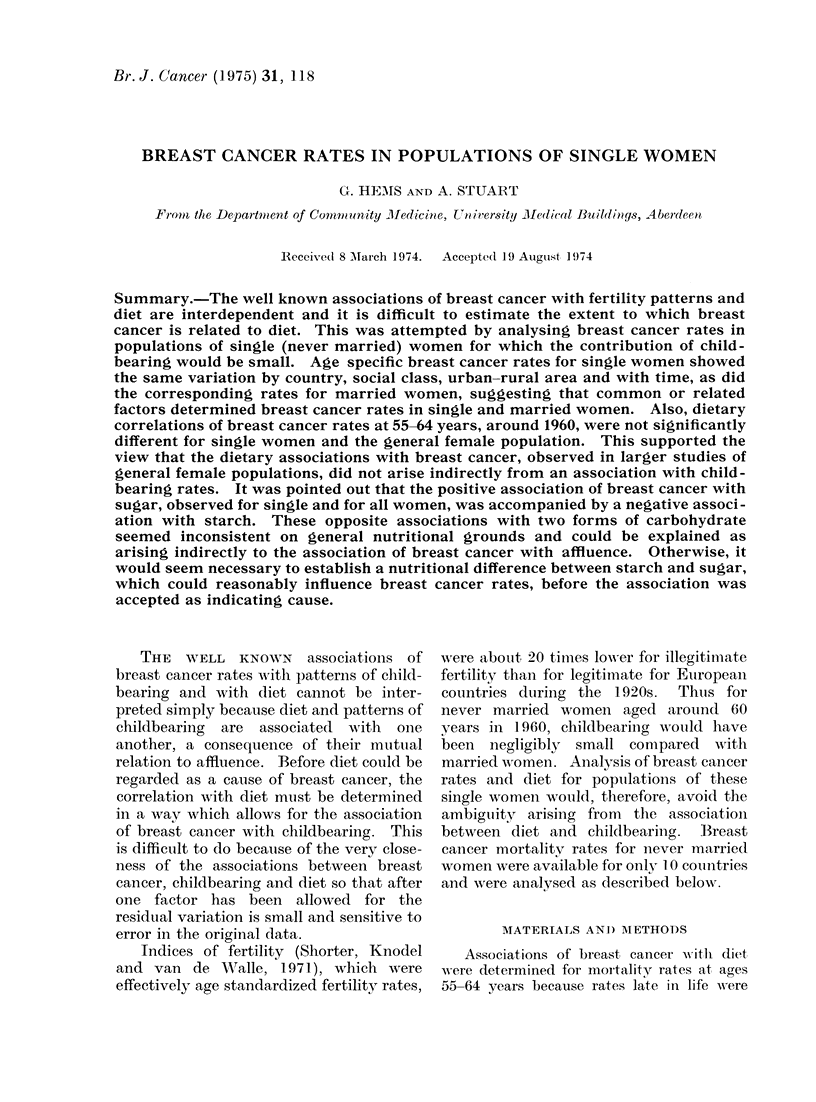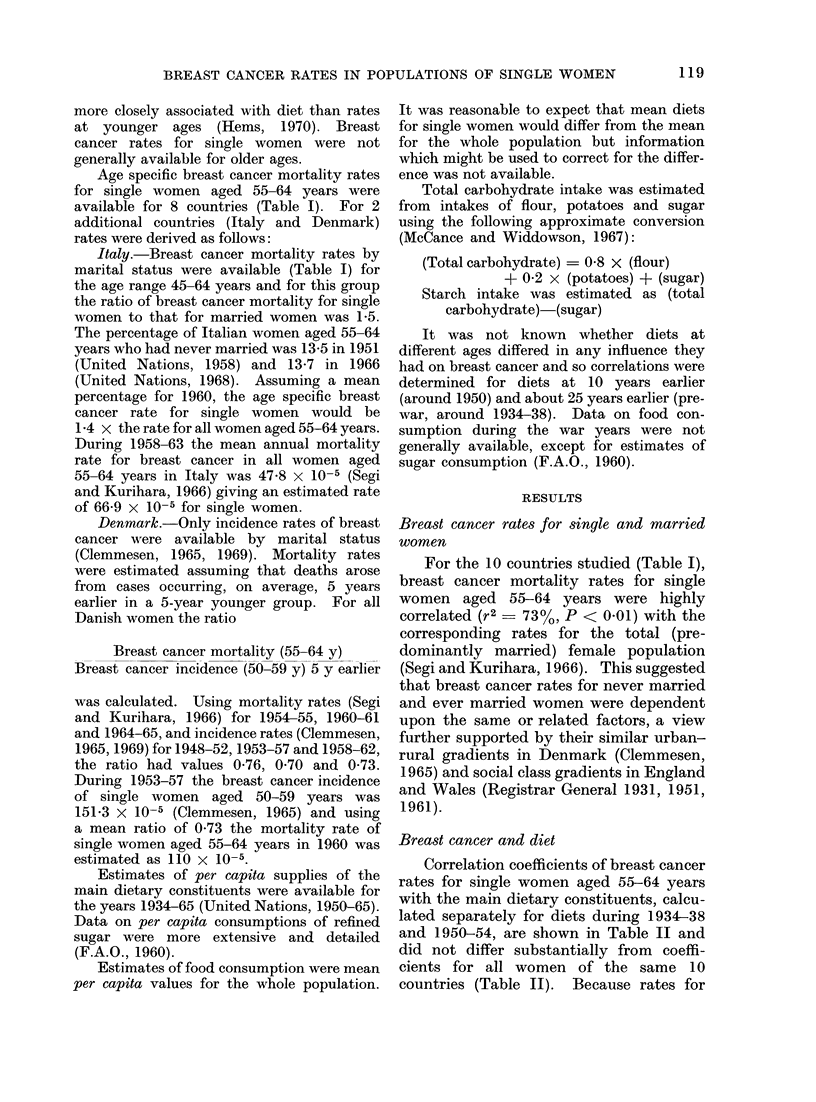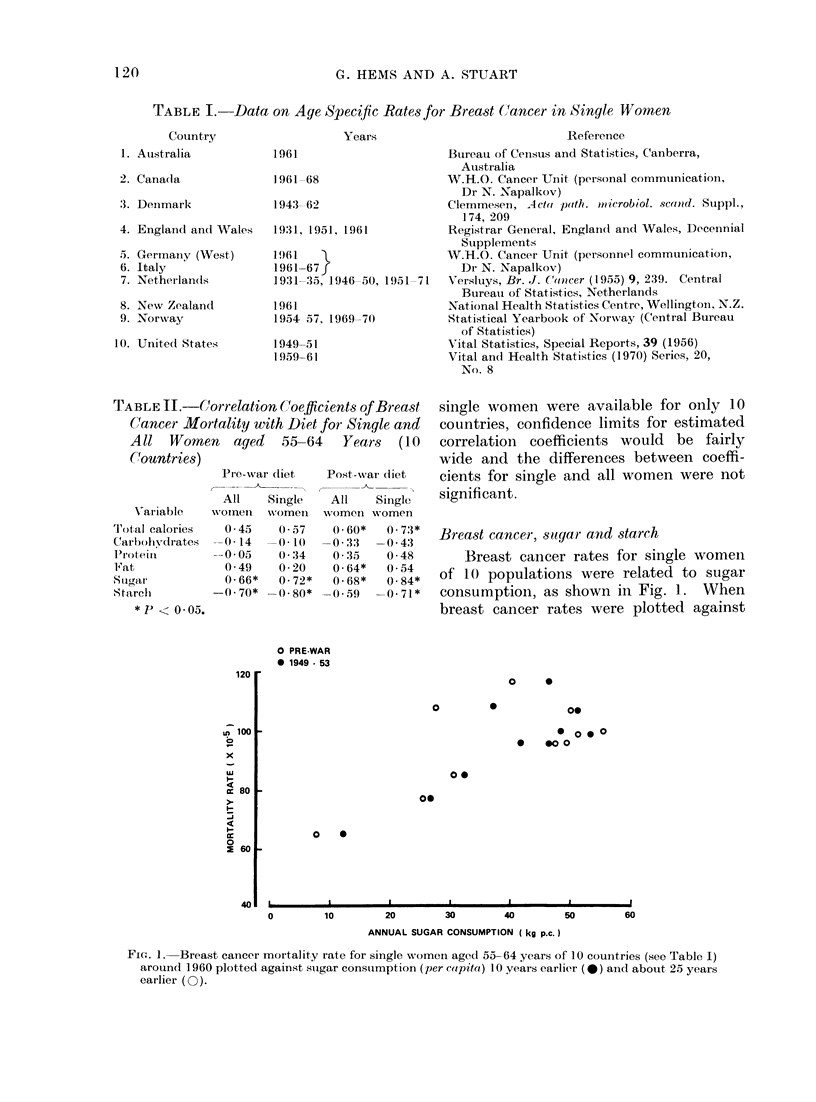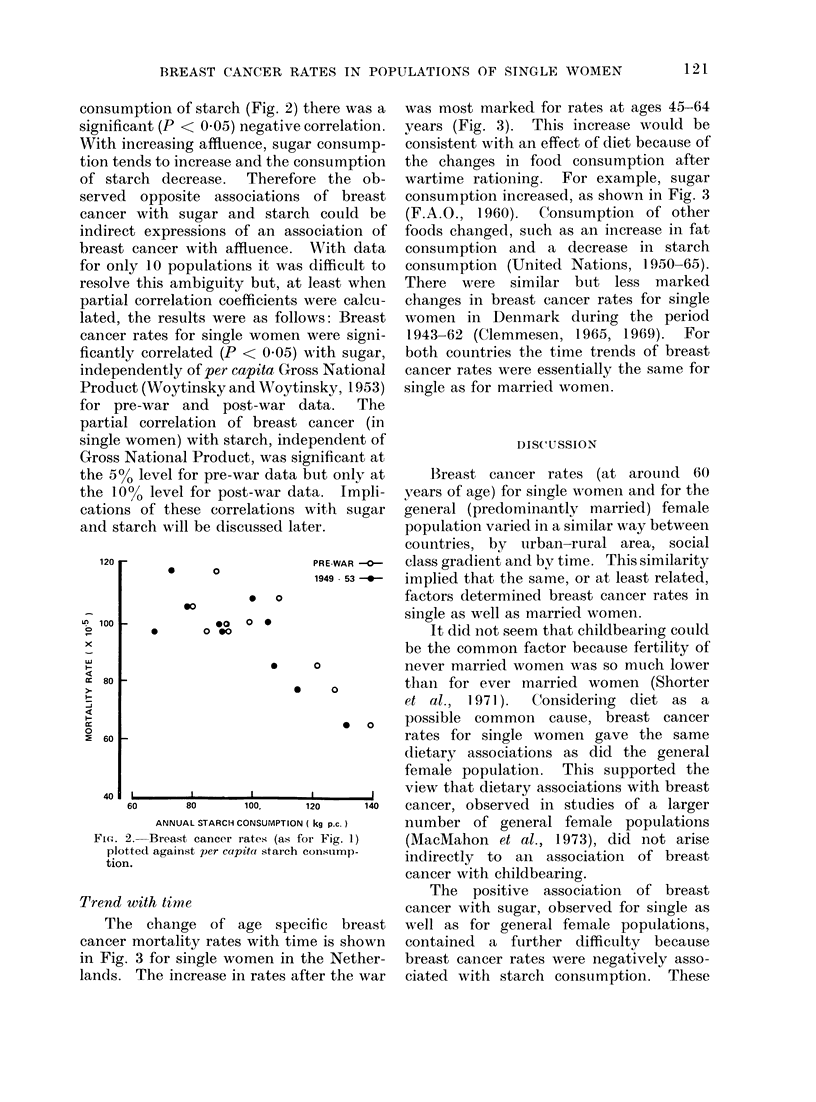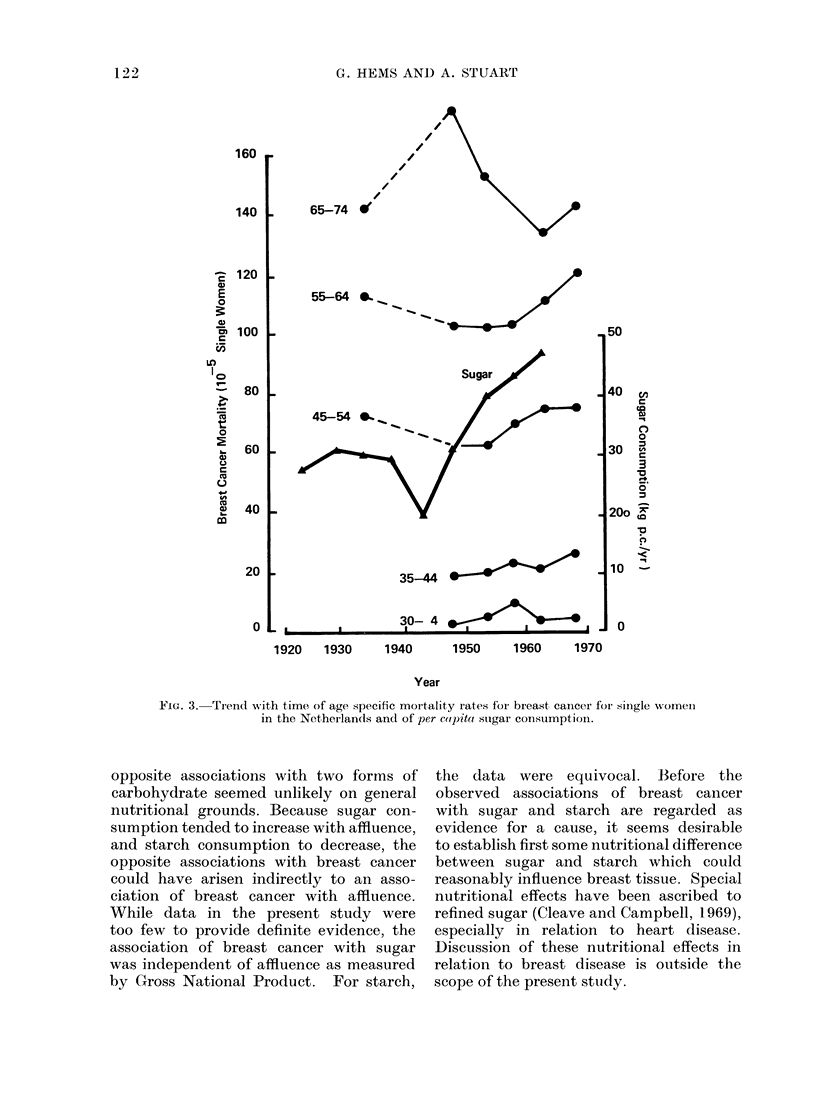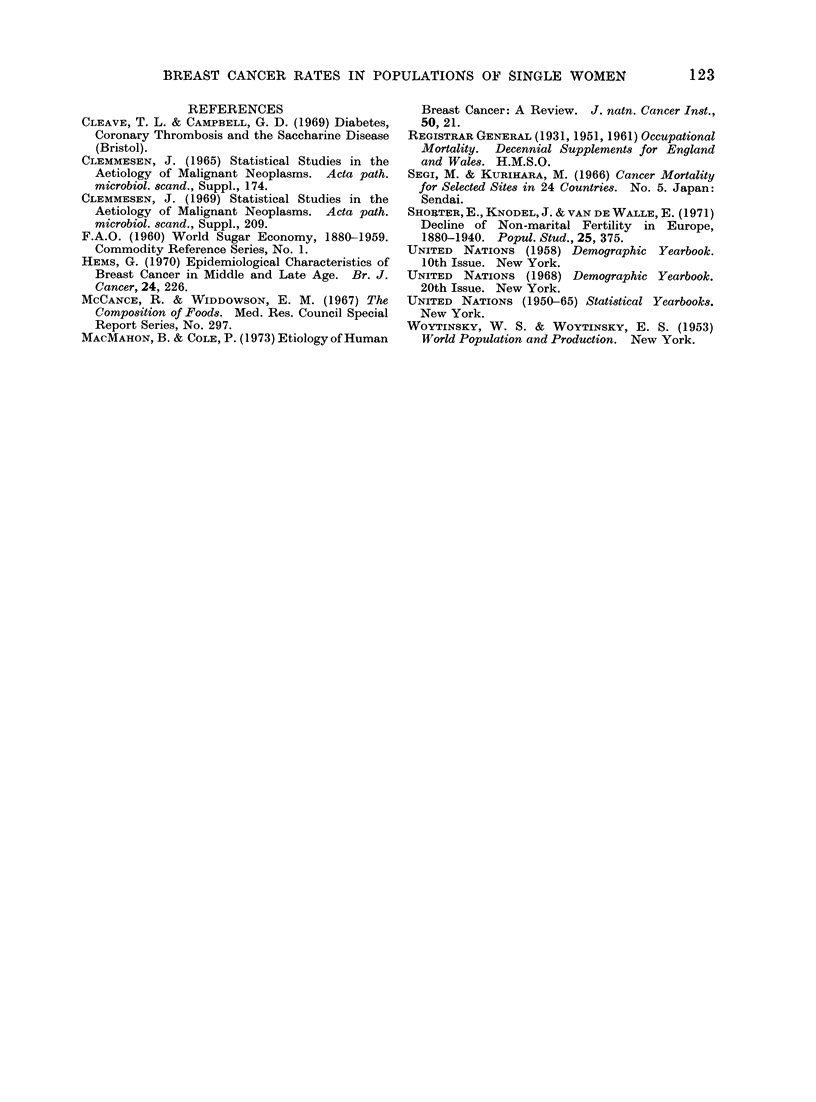# Breast cancer rates in populations of single women.

**DOI:** 10.1038/bjc.1975.14

**Published:** 1975-01

**Authors:** G. Hems, A. Stuart

## Abstract

The well known associations of breast cancer with fertility patterns and diet are interdependent and it is difficult to estimate the extent to which breast cancer is related to diet. This was attempted by analysing breast cancer rates in populations of single (never married) women for which the contribution of childbearing would be small. Age specific breast cancer rates for single women showed the same variation by country, social class, urban-rural area and with time, as did the corresponding rates for married women, suggesting that common or related factors determined breast cancer rates in single and married women. Also, dietary correlations of breast cancer rates at 55-64 years, around 1960, were not sifnificantly different for single women and the general female population. This supported the view that the dietary associations with breast cancer, observed in larger studies of general female populations, did not arise indirectly from an association with childbearing rates. It was pointed our that the positive association of breast cancer with sugar, observed for single and for all women, was accopanied by a negative association with starch. These opposite associations with two forms of varbohydrate seemed inconsistent on general nutritional grounds and could be explained as arising indirectly to the association of breast cancer with affluence. Otherwise, it would seem necessary to establish a nutritional difference between starch and sugar, which could reasonably influence breast cancer rates, before the association was accepted as indicating cause.


*Br. J. Cancer* (1975) **31**, 118

### BREAST CANCER RATES IN POPULATIONS OF SINGLE WOMEN

G. HEMS AND A. STUART

*From the Department of Community Medicine, University Medical Buildings, Aberdeen*

Received 8 March 1974. Accepted 19 August 1974

**Summary.—The well known associations of breast cancer with fertility patterns and diet are interdependent and it is difficult to estimate the extent to which breast cancer is related to diet. This was attempted by analysing breast cancer rates in populations of single (never married) women for which the contribution of child-bearing would be small. Age specific breast cancer rates for single women showed the same variation by country, social class, urban–rural area and with time, as did the corresponding rates for married women, suggesting that common or related factors determined breast cancer rates in single and married women. Also, dietary correlations of breast cancer rates at 55–64 years, around 1960, were not significantly different for single women and the general female population. This supported the view that the dietary associations with breast cancer, observed in larger studies of general female populations, did not arise indirectly from an association with child-bearing rates. It was pointed out that the positive association of breast cancer with sugar, observed for single and for all women, was accompanied by a negative association with starch. These opposite associations with two forms of carbohydrate seemed inconsistent on general nutritional grounds and could be explained as arising indirectly to the association of breast cancer with affluence. Otherwise, it would seem necessary to establish a nutritional difference between starch and sugar, which could reasonably influence breast cancer rates, before the association was accepted as indicating cause.**

THE WELL KNOWN associations of breast cancer rates with patterns of child-bearing and with diet cannot be interpreted simply because diet and patterns of childbearing are associated with one another, a consequence of their mutual relation to affluence. Before diet could be regarded as a cause of breast cancer, the correlation with diet must be determined in a way which allows for the association of breast cancer with childbearing. This is difficult to do because of the very closeness of the associations between breast cancer, childbearing and diet so that after one factor has been allowed for the residual variation is small and sensitive to error in the original data.

Indices of fertility (Shorter, Knodel and van de Walle, 1971), which were effectively age standardized fertility rates, were about 20 times lower for illegitimate fertility than for legitimate for European countries during the 1920s. Thus for never married women aged around 60 years in 1960, childbearing would have been negligibly small compared with married women. Analysis of breast cancer rates and diet for populations of these single women would, therefore, avoid the ambiguity arising from the association between diet and childbearing. Breast cancer mortality rates for never married women were available for only 10 countries and were analysed as described below.

### MATERIALS AND METHODS

Associations of breast cancer with diet were determined for mortality rates at ages 55–64 years because rates late in life were more closely associated with diet than rates at younger ages (Hems, 1970). Breast cancer rates for single women were not generally available for older ages.

Age specific breast cancer mortality rates for single women aged 55–64 years were available for 8 countries (Table I). For 2 additional countries (Italy and Denmark) rates were derived as follows:

*Italy.*—Breast cancer mortality rates by marital status were available (Table I) for the age range 45–64 years and for this group the ratio of breast cancer mortality for single women to that for married women was 1·5. The percentage of Italian women aged 55–64 years who had never married was 13·5 in 1951 (United Nations, 1958) and 13·7 in 1966 (United Nations, 1968). Assuming a mean percentage for 1960, the age specific breast cancer rate for single women would be 1·4 × the rate for all women aged 55–64 years. During 1958–63 the mean annual mortality rate for breast cancer in all women aged 55–64 years in Italy was $47 \cdot 8 \times 10^{-5}$ (Segi and Kurihara, 1966) giving an estimated rate of $66 \cdot 9 \times 10^{-5}$ for single women.

*Denmark.*—Only incidence rates of breast cancer were available by marital status (Clemmesen, 1965, 1969). Mortality rates were estimated assuming that deaths arose from cases occurring, on average, 5 years earlier in a 5-year younger group. For all Danish women the ratio

$$\frac{\text{Breast cancer mortality (55–64 y)}}{\text{Breast cancer incidence (50–59 y) 5 y earlier}}$$

was calculated. Using mortality rates (Segi and Kurihara, 1966) for 1954–55, 1960–61 and 1964–65, and incidence rates (Clemmesen, 1965, 1969) for 1948–52, 1953–57 and 1958–62, the ratio had values 0·76, 0·70 and 0·73. During 1953–57 the breast cancer incidence of single women aged 50–59 years was $151 \cdot 3 \times 10^{-5}$ (Clemmesen, 1965) and using a mean ratio of 0·73 the mortality rate of single women aged 55–64 years in 1960 was estimated as $110 \times 10^{-5}$.

Estimates of *per capita* supplies of the main dietary constituents were available for the years 1934–65 (United Nations, 1950–65). Data on *per capita* consumptions of refined sugar were more extensive and detailed (F.A.O., 1960).

Estimates of food consumption were mean *per capita* values for the whole population. It was reasonable to expect that mean diets for single women would differ from the mean for the whole population but information which might be used to correct for the difference was not available.

Total carbohydrate intake was estimated from intakes of flour, potatoes and sugar using the following approximate conversion (McCance and Widdowson, 1967):

$$\text{(Total carbohydrate)} = 0 \cdot 8 \times \text{(flour)} + 0 \cdot 2 \times \text{(potatoes)} + \text{(sugar)}$$

Starch intake was estimated as (total carbohydrate)—(sugar)

It was not known whether diets at different ages differed in any influence they had on breast cancer and so correlations were determined for diets at 10 years earlier (around 1950) and about 25 years earlier (prewar, around 1934–38). Data on food consumption during the war years were not generally available, except for estimates of sugar consumption (F.A.O., 1960).

### RESULTS

### *Breast cancer rates for single and married women*

For the 10 countries studied (Table I), breast cancer mortality rates for single women aged 55–64 years were highly correlated ($r^2 = 73\%$, $P < 0 \cdot 01$) with the corresponding rates for the total (predominantly married) female population (Segi and Kurihara, 1966). This suggested that breast cancer rates for never married and ever married women were dependent upon the same or related factors, a view further supported by their similar urban–rural gradients in Denmark (Clemmesen, 1965) and social class gradients in England and Wales (Registrar General 1931, 1951, 1961).

### *Breast cancer and diet*

Correlation coefficients of breast cancer rates for single women aged 55–64 years with the main dietary constituents, calculated separately for diets during 1934–38 and 1950–54, are shown in Table II and did not differ substantially from coefficients for all women of the same 10 countries (Table II). Because rates for

TABLE I.—*Data on Age Specific Rates for Breast Cancer in Single Women*

| Country | Years | Reference |
|---|---|---|
| 1. Australia | 1961 | Bureau of Census and Statistics, Canberra, Australia |
| 2. Canada | 1961–68 | W.H.O. Cancer Unit (personal communication, Dr N. Napalkov) |
| 3. Denmark | 1943–62 | Clemmesen, *Acta path. microbiol. scand.* Suppl., 174, 209 |
| 4. England and Wales | 1931, 1951, 1961 | Registrar General, England and Wales, Decennial Supplements |
| 5. Germany (West) | 1961 } | W.H.O. Cancer Unit (personnel communication, Dr N. Napalkov) |
| 6. Italy | 1961–67 } | |
| 7. Netherlands | 1931–35, 1946–50, 1951–71 | Versluys, *Br. J. Cancer* (1955) **9**, 239. Central Bureau of Statistics, Netherlands |
| 8. New Zealand | 1961 | National Health Statistics Centre, Wellington, N.Z. |
| 9. Norway | 1954–57, 1969–70 | Statistical Yearbook of Norway (Central Bureau of Statistics) |
| 10. United States | 1949–51 | Vital Statistics, Special Reports, **39** (1956) |
| | 1959–61 | Vital and Health Statistics (1970) Series, 20, No. 8 |

TABLE II.—*Correlation Coefficients of Breast Cancer Mortality with Diet for Single and All Women aged 55–64 Years (10 Countries)*

| | Pre-war diet | | Post-war diet | |
|---|---|---|---|---|
| Variable | All women | Single women | All women | Single women |
| Total calories | 0·45 | 0·57 | 0·60* | 0·73* |
| Carbohydrates | −0·14 | −0·10 | −0·33 | −0·43 |
| Protein | −0·05 | 0·34 | 0·35 | 0·48 |
| Fat | 0·49 | 0·20 | 0·64* | 0·54 |
| Sugar | 0·66* | 0·72* | 0·68* | 0·84* |
| Starch | −0·70* | −0·80* | −0·59 | −0·71* |

$*P < 0·05$.

single women were available for only 10 countries, confidence limits for estimated correlation coefficients would be fairly wide and the differences between coefficients for single and all women were not significant.

*Breast cancer, sugar and starch*

Breast cancer rates for single women of 10 populations were related to sugar consumption, as shown in Fig. 1. When breast cancer rates were plotted against

FIG. 1.—Breast cancer mortality rate for single women aged 55–64 years of 10 countries (see Table I) around 1960 plotted against sugar consumption (*per capita*) 10 years earlier (●) and about 25 years earlier (○).

consumption of starch (Fig. 2) there was a significant ($P < 0{\cdot}05$) negative correlation. With increasing affluence, sugar consumption tends to increase and the consumption of starch decrease. Therefore the observed opposite associations of breast cancer with sugar and starch could be indirect expressions of an association of breast cancer with affluence. With data for only 10 populations it was difficult to resolve this ambiguity but, at least when partial correlation coefficients were calculated, the results were as follows: Breast cancer rates for single women were significantly correlated ($P < 0{\cdot}05$) with sugar, independently of *per capita* Gross National Product (Woytinsky and Woytinsky, 1953) for pre-war and post-war data. The partial correlation of breast cancer (in single women) with starch, independent of Gross National Product, was significant at the 5% level for pre-war data but only at the 10% level for post-war data. Implications of these correlations with sugar and starch will be discussed later.

FIG. 2.—Breast cancer rates (as for Fig. 1) plotted against *per capita* starch consumption.

*Trend with time*

The change of age specific breast cancer mortality rates with time is shown in Fig. 3 for single women in the Netherlands. The increase in rates after the war was most marked for rates at ages 45–64 years (Fig. 3). This increase would be consistent with an effect of diet because of the changes in food consumption after wartime rationing. For example, sugar consumption increased, as shown in Fig. 3 (F.A.O., 1960). Consumption of other foods changed, such as an increase in fat consumption and a decrease in starch consumption (United Nations, 1950–65). There were similar but less marked changes in breast cancer rates for single women in Denmark during the period 1943–62 (Clemmesen, 1965, 1969). For both countries the time trends of breast cancer rates were essentially the same for single as for married women.

### DISCUSSION

Breast cancer rates (at around 60 years of age) for single women and for the general (predominantly married) female population varied in a similar way between countries, by urban–rural area, social class gradient and by time. This similarity implied that the same, or at least related, factors determined breast cancer rates in single as well as married women.

It did not seem that childbearing could be the common factor because fertility of never married women was so much lower than for ever married women (Shorter *et al.*, 1971). Considering diet as a possible common cause, breast cancer rates for single women gave the same dietary associations as did the general female population. This supported the view that dietary associations with breast cancer, observed in studies of a larger number of general female populations (MacMahon *et al.*, 1973), did not arise indirectly to an association of breast cancer with childbearing.

The positive association of breast cancer with sugar, observed for single as well as for general female populations, contained a further difficulty because breast cancer rates were negatively associated with starch consumption. These

FIG. 3.—Trend with time of age specific mortality rates for breast cancer for single women in the Netherlands and of *per capita* sugar consumption.

opposite associations with two forms of carbohydrate seemed unlikely on general nutritional grounds. Because sugar consumption tended to increase with affluence, and starch consumption to decrease, the opposite associations with breast cancer could have arisen indirectly to an association of breast cancer with affluence. While data in the present study were too few to provide definite evidence, the association of breast cancer with sugar was independent of affluence as measured by Gross National Product. For starch, the data were equivocal. Before the observed associations of breast cancer with sugar and starch are regarded as evidence for a cause, it seems desirable to establish first some nutritional difference between sugar and starch which could reasonably influence breast tissue. Special nutritional effects have been ascribed to refined sugar (Cleave and Campbell, 1969), especially in relation to heart disease. Discussion of these nutritional effects in relation to breast disease is outside the scope of the present study.

REFERENCES

CLEAVE, T. L. & CAMPBELL, G. D. (1969) Diabetes, Coronary Thrombosis and the Saccharine Disease (Bristol).

CLEMMESEN, J. (1965) Statistical Studies in the Aetiology of Malignant Neoplasms. *Acta path. microbiol. scand.*, Suppl., 174.

CLEMMESEN, J. (1969) Statistical Studies in the Aetiology of Malignant Neoplasms. *Acta path. microbiol. scand.*, Suppl., 209.

F.A.O. (1960) World Sugar Economy, 1880–1959. Commodity Reference Series, No. 1.

HEMS, G. (1970) Epidemiological Characteristics of Breast Cancer in Middle and Late Age. *Br. J. Cancer*, **24**, 226.

MCCANCE, R. & WIDDOWSON, E. M. (1967) *The Composition of Foods*. Med. Res. Council Special Report Series, No. 297.

MACMAHON, B. & COLE, P. (1973) Etiology of Human Breast Cancer: A Review. *J. natn. Cancer Inst.*, **50**, 21.

REGISTRAR GENERAL (1931, 1951, 1961) *Occupational Mortality. Decennial Supplements for England and Wales*. H.M.S.O.

SEGI, M. & KURIHARA, M. (1966) *Cancer Mortality for Selected Sites in 24 Countries*. No. 5. Japan: Sendai.

SHORTER, E., KNODEL, J. & VAN DE WALLE, E. (1971) Decline of Non-marital Fertility in Europe, 1880–1940. *Popul. Stud.*, **25**, 375.

UNITED NATIONS (1958) *Demographic Yearbook*. 10th Issue. New York.

UNITED NATIONS (1968) *Demographic Yearbook*. 20th Issue. New York.

UNITED NATIONS (1950–65) *Statistical Yearbooks*. New York.

WOYTINSKY, W. S. & WOYTINSKY, E. S. (1953) *World Population and Production*. New York.